# Minimal representations of possibility at age 3

**DOI:** 10.1073/pnas.2207499119

**Published:** 2022-12-19

**Authors:** Brian Leahy, Michael Huemer, Matt Steele, Stephanie Alderete, Susan Carey

**Affiliations:** ^a^Harvard University, Cambridge, MA 02138; ^b^ University of Salzburg, 5020 Salzburg, Austria

**Keywords:** conceptual development, logical concepts, possibility concepts

## Abstract

Young children do not always consider alternative possibilities when planning. Suppose a prize is hidden in a single occluded container and another prize is hidden in an occluded pair. If given a chance to choose one container and receive its contents, choosing the singleton maximizes expected reward because each member of the pair might be empty. Yet, 3-y-olds choose a member of the pair almost half the time. Why don’t they maximize expected reward? Three studies provide evidence that 3-y-olds do not deploy possibility concepts like MIGHT, which would let them represent that each container in the pair might and might not contain a prize. Rather, they build an overly specific model of the situation that correctly specifies that the singleton holds a prize while inappropriately specifying which member of the pair holds a prize and which is empty. So, when asked to choose a container, they see two equally good options. This predicts approximately 50% choice of the singleton, observed in studies 1 and 3. But when asked to throw away a container so that they can receive the remaining contents (study 2), they mostly throw away a member of the pair. The full pattern of data is expected if children construct overly specific models. We discuss whether 3-year-olds lack possibility concepts or whether performance demands prevent deployment of them in our tasks.

Young children make surprisingly unwise decisions in the face of multiple possibilities (1). Consider the three-container task (Fig. 1). Children see a stage with three containers, organized into a pair and a singleton. The singleton is occluded, and a prize is hidden there. Next, the pair is occluded, and a prize is hidden there. When all three containers are visible, children are told that they have one chance to pick one container to get a prize, so they should choose the one that is sure to have a prize. The wise decision—i.e., the decision that maximizes their expected reward—is to choose the singleton. Older 2-y-olds (like chimpanzees) pick wisely 50% of the time, and 3-y-olds 60% of the time (2–4). Participants are not guessing at random (chance is .33), yet they are far from maximizing expected reward.

**Fig. 1.**
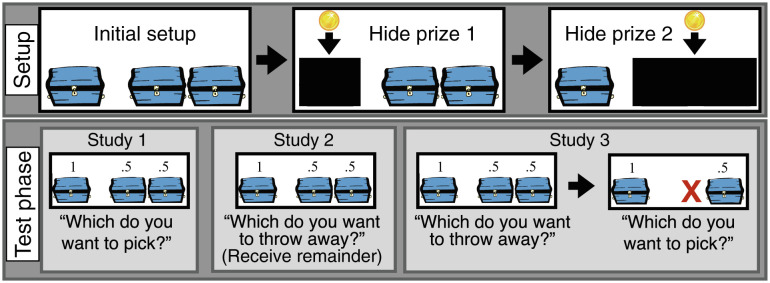
Test trials for three studies. All three studies have a common setup phase (*Top* row). The question posed in the test phase differs across the three studies (*Bottom* row). The red “X” in the “Which do you pick?” question of study 3 is visible on the screen and indicates the location of the chest that was thrown away. Numbers above chests in the test phase indicate the probability that the chest holds a prize.

What computational process yields this highly replicable behavior—better than chance, but far from maximizing? One proposal is that chimpanzees, older 2-y-olds, and most 3-y-olds deploy minimal representations of possibility (1). They infer the location of the prize in the singleton, as there is only one place for it to be. They simulate the other prize going into one member of the pair. But instead of bearing in mind that that outcome is merely possible, they take the simulated location to be the fact of the matter. Thus, they have a belief about each prize’s location: One prize in the singleton, and another prize in the simulated member of the pair. The child deploys no possibility concepts—that is, representational indicators (markers, symbols) of mere possibility—to distinguish the simulated belief from the inferred belief. The phrase, “Minimal representation of possibility” aims to express that the represented state, which is generated by simulation, is merely possible. But its mere possibility is not marked in the representation. This computational process yields 50% choice of the singleton: Since the child has a belief about each prize’s location, she chooses at random between those two locations.

However, 50% choice of a member of the pair could arise in many ways. Children might have a side bias, might focus on only one prize during the setup phase, or may be choosing between the two sides at random. These low-level strategies engage neither minimal representations of possibility nor possibility concepts; they do not even require working memory models of one prize on each side. Here, we report three studies (depicted in Fig. 1) that adjudicate between the hypotheses that children deploy possibility concepts, that they deploy minimal representations of possibility, and that they deploy these low-level strategies in the three-container task. The predictions of each hypothesis appear in Fig. 2*A* and are described within the presentation of each study.

**Fig. 2.**
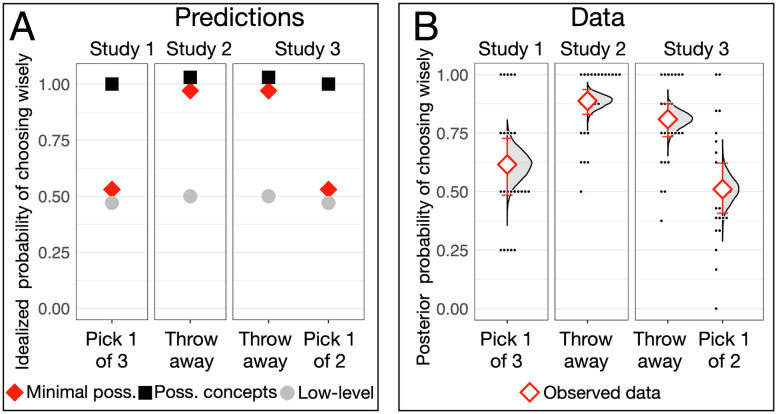
Idealized predictions and data. (*A*) Idealized predictions of three hypotheses. Points are dodged vertically to avoid overlap. Points show predictions from deploying minimal representations of possibility (red diamonds), possibility concepts (black squares), and low-level strategies (gray circles). (*B*) Observed data and estimated probability of a wise decision. Study 1: Picking the singleton cup. Study 2: Throwing away from the pair. Study 3: Throwing away from the pair and then (eliminating trials where participants had thrown away the singleton cup) picking the singleton cup. Red points are posterior medians. Error bars are 95% CIs (highest-density intervals). Distributions are Bayesian posteriors. Small dots are individual participants’ proportions of wise decisions.

## Study 1: Pick 1 of 3

Study 1 tested whether an online three-container task (Fig. 1) replicated in-lab findings that 3-y-olds choose wisely 60% of the time (2, 3). After training, twenty-four 3-y-olds (mean age = 3.45, range = 3.01–3.94, 13 f) saw four test trials, choosing one chest to receive its contents.

Children did not choose among the three chests at chance: Bayesian generalized linear mixed model (GLMM), estimated probability of choosing wisely .62, 95% CI [.48, .73], probability > .33 ≈ 1. This replicated published three-container tasks: .61 in ref. 2 and .62 in ref. 3; *SI Appendix*. All of these results are expected if most children deploy minimal representations of possibility or equally if most children deploy the low-level strategies detailed above. Study 2 adjudicated between these hypotheses.

## Study 2: Throw Away

In study 2, we taught twenty-four 3-y-olds (mean age = 3.63, range = 3.14–3.94, 10 f) that when they threw a chest away, they received the contents of all remaining chests. In each of the eight test trials, they were reminded to try to get two coins and then asked which chest they wanted to throw away. Children deploying possibility concepts or minimal representations of possibility should reliably throw away a chest from the pair: a chest that merely might hold a coin or the chest that they believe is empty, respectively (Fig. 2*A*). Children who choose sides randomly or focus on only one coin randomly should throw away from the pair about half of the time—less than chance (.67). The estimated probability of throwing away from the pair was .89, 95% CI [.83, .94]; probability > .67 ≈ 1 (Fig. 2*B* and *SI Appendix*). Moreover, if children are choosing a side or a coin at random, there should be no difference between the Throw Away task and the Pick 1 of 3 task. If children deploy minimal representations of possibility, performance on the Throw Away task should be better than Pick 1 of 3: They throw away the chest they take to be empty, which is always a member of the pair. This was observed (log odds ratio (log OR) 1.60, 95% CI [0.87, 2.36]; *SI Appendix*). These results rule out the low-level strategy hypothesis. They are as expected if children deploy minimal representations of possibility. They are also expected if prompting 3-y-olds to think about which container is empty somehow elicited the deployment of possibility concepts. Study 3 adjudicated between these hypotheses.

## Study 3: Throw Away and Pick 1 of 2

Study 3 gave twenty-four 3-y-olds (mean age = 3.63, range = 3.04–3.96, 17 f) eight trials in which they were asked to throw away a chest that they do not want and then to choose one of the two remaining chests. Children threw away from the pair more often than expected by chance (estimated probability of throwing away from the pair: .81, 95% CI [.73, .88]; probability > .67 ≈ 1) and made wise decisions on Throw Away more often than in Pick 1 of 3 in study 1 (log OR 0.98, 95% CI [0.31, 1.71]), replicating study 2. These results are expected if children deploy minimal representations of possibility and also if they deploy possibility concepts. The Pick 1 of 2 question tested these hypotheses (Fig. 2*A*). Participants who deploy possibility concepts should choose wisely (the singleton) because the remaining member of the pair merely might hold a coin. Participants who deploy minimal representations of possibility should choose wisely half the time since they believe that both remaining chests hold a coin. Only trials where children were choosing between the singleton and a member of the pair (79% of trials) were included in this analysis because our question is whether children prefer the singleton over the remaining member of the pair. Children chose wisely (the singleton) at chance levels (chance = .50; estimated probability of choosing wisely .51, 95% CI [.41, .62]). The hypothesis that children chose the singleton chest 50% of the time is more likely than 99% of the hypotheses within the 95% CI; *SI Appendix*. Moreover, participants who deploy minimal representations of possibility should choose wisely less often on Pick 1 of 2 than on Throw Away (Fig. 2*A*). Indeed, this was so (log OR −1.41, 95% CI [−1.94, −0.91]).

The full pattern of responses (Fig. 2*B*) fits best with the minimal representations of possibility hypothesis (Fig. 2*A*). Only that hypothesis predicts the observed differences between throw away and pick 1 trial types.

## Departures from Quantitative Predictions

The hypothesis that children deploy minimal representations of possibility makes quantitative predictions in each trial type, several of which are not met. See *SI Appendix*, section II for discussion of the deviation from 100% wise decisions on the Throw Away trials.

In Pick 1 of 3, 3-y-olds choose wisely 60% of the time, not 50%. The expected 50% performance is observed in older 2-y-olds (46% in ref. (2), 48% in ref. 3) and in chimpanzees (51% in ref. 4). The observed 60% performance of 3-y-olds can be explained if most children deployed minimal representations of possibility and some deployed possibility concepts, most likely in a 80%/20% mixture (.8 * .5 + .2 * 1 = .60, the observed mean). We found that the observed distribution of individual participants’ proportion of wise decisions was almost identical to the distribution expected under this hypothesis (exact multinomial test, *P* = .90). Small dots in Fig. 2*B*, study 1, display this distribution. It reveals a textbook binomial distribution centered on .5, save that there are too many participants who performed perfectly. See *SI Appendix*, section III for statistical details.

This analysis strategy allows us to test an additional hypothesis that could yield the observed mean. We have already rejected the hypothesis that all 3-y-olds choose at random since participants choose wisely on the Pick 1 of 3 measure much more than a third of the time. But if the population were a 60%/40% mixture of children who choose one of three chests at random and children who always choose wisely, we would expect the observed 60% wise decisions (.6 * .33 + .4 * 1 = .60). However, the observed distribution of proportions (small dots in Fig. 2*B*, Study 1) differs significantly from the distribution expected under this hypothesis (exact multinomial test, *P* = .001). See *SI Appendix*, section III for statistical details.

## Discussion

Children are not bringing possibility concepts to bear on these tasks, nor do they deploy the obvious low-level strategies that would generate approximately 50% choice of the singleton. As one participant helpfully explained one of his throw-away decisions, “A coin went in this one (pointing to the singleton), and a coin went in this one (one of the pair), so throw away that one (the remaining chest from the pair).” These three studies, and this explicit explanation, provide strong evidence that 3-y-olds deploy minimal representations of possibility in these tasks.

The failure of 3-y-olds to deploy possibility concepts on three-container tasks coincides with failures at this age to deploy possibility concepts in other action planning tasks and in the comprehension of the language of possibility.

### Other Action Planning Tasks.

When a reward is dropped into a tube that forks at the bottom, 2.5-y-olds who want to catch the reward almost always cover just one exit, missing the reward about half of the time (5). This is as predicted if they deploy minimal representations of possibility. They fail to deploy possibility concepts, as they show no insight that the reward might come out of either exit. Not until 4 years of age do children spontaneously and consistently cover both exits (6).

The “three-exit task” eliminated working memory demands of the three-container task by making everything the child needs to know visible and salient while they plan their action (7). Participants tried to catch marbles in a cup. Two marbles were simultaneously rolled into two fully visible channels. One channel forked at the bottom so that participants could not anticipate where its marble would come out. The other channel did not fork; its marble followed a determinate path. Thus, there is one exit where a marble will come out (the unforked channel, like the singleton container), and there are two exits where a marble merely might come out (each branch of the forked channel, like the pair in the three-container task). Since the cup could cover only one exit, the wise place to put it is under the nonforked channel. This task makes the same logical reasoning demands as the three-container task, but very different executive function demands. Children chose wisely at identical rates on the three-exit and three-container tasks (older 2-y-olds 49% wise decisions; 3-y-olds 61% wise decisions), indicating that young children most likely deploy minimal representation of possibility in both.

### Modal Language.

By age 2 or 3, children use words like “maybe,” “can,” and “have to” that express possibility and necessity in adult language (8). But systematic studies of their comprehension show that children begin to analyze this language with adult meaning only after their fourth birthday. While most 4-y-olds correctly judge that possible events can happen and impossible events cannot, they incorrectly judge that merely possible events have to happen (9). This pattern is expected if children answer each question on the basis of a single simulation, checking whether anything prevents the event that the question asks about and answering “no” if that event is prevented, “yes” otherwise; i.e., using the resources of minimal representations of possibility. To correctly answer questions about what has to happen, the child has to recognize when there are multiple possible outcomes for a single event and hence that merely possible events do not have to happen. The observed pattern of errors is expected if children are answering all modal questions on the basis of a single simulation.

Jointly, all the above failures raise the possibility that children do not deploy possibility concepts—symbolic markers that mark propositions as merely possible—because they do not have them to deploy. This hypothesis has not been ruled out by existing research (1).

Taking this hypothesis seriously raises questions for further research. It is possible that 3-y-olds do have possibility concepts, but diverse performance issues prevent deployment of possibility concepts in each of these tasks. If so, we should be able to develop novel tasks that reveal possibility concepts in 3-y-olds and characterize the performance demands that prevent 3-y-olds from deploying possibility concepts. Indeed, the three-exit task was an attempted step in this line of research but instead established that working memory demands were not masking children’s possibility concepts. Further research should continue exploring parallels in the developmental course of possibility concepts across tasks with diverse performance demands, including seeking within-child correlations and controlling for age and executive function.

If the evidence were to favor the hypothesis that most 3-y-olds lack possibility concepts, this would raise a daunting challenge: specifying a mechanism that supports the development of possibility concepts out of available representational and computational capacities that lack symbols for possibility. Are minimal representations of possibilities precursors to possibility concepts, in the sense of playing a causal role in their acquisition? If children initially analyze words like “maybe” and “can” in terms of minimal representations of possibility, perhaps the full pattern of use of modal words plays a role in acquiring the concept of possibility. Further research should explore the details of how children learn modal language as well as the relations between its acquisition and performance on action planning tasks that probe possibility concepts.

## Materials and Methods

### Materials and Procedure.

Tasks were presented via Zoom. Children indicated their choices by pointing; caregivers communicated the child’s choice. In training, children learned to make wise decisions—whether picking a chest, throwing away a chest, or throwing away a chest and then picking one—in full knowledge of which chests held coins and which were empty. Test trials are described in Fig. 1. See *SI Appendix* for complete participant information, procedure, and counterbalancing. Ethical approval for all studies was granted by the Harvard Institutional Review Board. Caregivers provided informed consent prior to each study. Children gave verbal assent.

### Analysis Strategy.

Data were analyzed with a Bayesian GLMM with a random intercept for participant id. We used default priors. The DV was the participant’s choice: Did they choose wisely (1) or not (0)? The IV was trial type [a factor with four levels: Pick 1 of 3, Throw Away, Throw Away (study 3), and Pick 1 of 2]. Statistical methods and additional models are detailed in *SI Appendix*.

### Open Science Practices.

Pregistration for study 3 is at https://aspredicated.org/aq2ct.pdf.

## Supplementary Material

Appendix 01 (PDF)

## Data Availability

Anonymized [Script, data, and code] data have been deposited in [dataverse.harvard.edu/] (10.7910/DVN/PD6RAG).
