# Supplementary material for: Minimal representations of possibility at age 3
Source: Proc Natl Acad Sci U S A. 2022 Dec 19;119(52):e2207499119. doi: 10.1073/pnas.2207499119 (PMC9907095; doi:10.1073/pnas.2207499119)
Supplement: Supplementary file 1 — Appendix 01 (PDF) [file pnas.2207499119.sapp.pdf]

## Supporting Information: Minimal Representations of Possibility at Age 3

### Participants and exclusions

Our final sample included 72 3-year-olds, 24 per study (mean 3.57, range 3.01-3.96, 40 female). Sample sizes were chosen in advance, matched to the sample size in Mody and Carey (1). Children were recruited by email from a database of middle-class families from Cambridge, MA. Of the 61 families who chose to self-identify by race, 15% identified as Asian, 80% identified as White, and 5% identified as Other. Of the 60 families who chose to self-identify by ethnicity, 3% identified as Hispanic or Latino and 97% identified as not Hispanic or Latino. Caregivers provided informed consent prior to each study, and were assured that they could withdraw from participation at any time. Children gave verbal assent before starting. Families received a $5 Amazon gift card for participation.

An additional 19 children were tested but excluded because they failed to complete the experiment (n = 6) or they made two consecutive errors in the same training trial type (n = 13). Exclusion criteria were set in advance and preregistered for Study 3. All children participated in the game and received the gift card.

### Shared features of all studies

The child sat with a caregiver and communicated with the experimenter via Zoom. The stimuli were delivered on a slideshow controlled by the experimenter. Slideshows had a pirate theme, where children searched for gold coins in treasure chests. The child chose a chest by pointing. Caregivers communicated children's points to the experimenter.

Each of the three studies had a training phase where we taught children to make wise decisions under conditions of complete knowledge: Whenever they had to make a choice, they knew which chests held coins and which were empty. They were trained to open chests that held coins and to throw away empty chests, as the case may be. The question of interest was how they would transfer this training to a situation of incomplete knowledge, where there are two possible locations for one of the coins.

In all training trial types, if a child opened an empty chest, correction was provided and that trial was repeated. If a child made two consecutive errors (despite correction on the first), their data were excluded.

Whenever a chest that held a coin opened, the coin flew to the center of the screen, spun, and bounced off-screen accompanied by a fun, reinforcing jingle. Whenever an empty chest opened a 'disappointed' jingle played.

### Study 1: Pick 1 of 3

### Participants

Participants were 24 3-year-old children (mean = 3.45 years, range = 3.01-3.94 years, 13 female). Data from two additional children were excluded for making two consecutive errors in the two-chest training (see below).

### Procedure

We sought to replicate the in-lab finding that 3-year-olds pick wisely (i.e., pick the singleton chest) on 60% of Pick 1 of 3 trials in the 3-container task (1,2).

**Warmup.** In the warmup, six differently-colored treasure chests appeared on-screen. The child was asked to name the color of each chest. The goal of the task was interactivity, not accuracy, so any answer was accepted.

**Training phase** In the training phase children learned to open a chest and find a coin, under conditions of perfect information: Every time they chose a chest, they knew the location of every coin.

***Training phase 1: Single chest.*** The first training trial familiarized children with the goal of the game: to open a chest that held a coin. This trial started with one chest on the screen. A coin appeared above it. The chest opened and the coin moved into the chest, which then closed. The child was asked where the coin had been hidden. Once the child indicated the chest, it opened and revealed the coin.

***Training phase 2: Two chests.*** In the next training trial two chests appeared on screen. Then a coin appeared above the chests. The chests opened, the coin moved into one of the chests, and the chests closed. Then the child was asked which chest she or he wanted to open. The contents of the selected chest were then revealed. All children saw at least two trials.

***Training phase 3: Demonstration of appropriate reasoning.*** In these trials we told children how to solve the problem–to pick a chest that was sure to hold a coin–without teaching them to pick a singleton chest. Two pairs of chests appeared. One pair was occluded by a pirate flag. Two coins appeared, one above each occluded chest. These coins simultaneously descended directly downward so that children could tell that one coin went into each occluded chest. The occluder was removed, and the other pair was occluded. One coin appeared above the space between the two occluded chests; it descended and was hidden behind the flag in one of the two chests. The occluder was removed. It was not clear which of those two chests held the coin. After all coins were hidden, the experimenter indicated the side where two coins were hidden and told the child that if he picked a chest on that side he could be certain that he would win a coin. But if he picked a chest on the other side, he could *not* be certain that he would win a coin. The experimenter then opened a chest on the side with two coins. Thus he told children how to solve the problem and illustrated the solution, without modeling a behavior that could be copied in the 3-chest test trials. The demonstration was repeated, changing only which pair of chests held two coins. Three variables were counterbalanced between participants: the side where coins were hidden first (L/R), the side first indicated and discussed by the experimenter (L/R), and the location of the two coin side in the first demonstration (L/R).

**Test phase.** Two sets of chests appeared: a singleton and a pair (main text, Fig. 1). One set was occluded and a coin was hidden, and the occluder disappeared. Then the other set was occluded and a second coin was hidden there, and the occluder disappeared. The child was asked which chest they were “really sure” held a coin. The chosen chest opened and revealed its contents. Children saw four trials. Three variables were counterbalanced between participants: the location of the singleton chest in the first trial (L/R), the positioning of the coin in the pair

(LRRL / RLLR), and the side where coins were hidden first (L/R). The side (L/R) where coins were hidden first was constant within participants across trials. The side of the singleton and pair swapped sides each trial.

**Study 2: Throw Away**

**Participants**

Participants were 24 3-year-olds (mean = 3.63, range = 3.14-3.94, 10 female). Data from eight additional children were excluded: three failed to complete the study, four made consecutive errors in the three-chest training, and one made consecutive errors in the flag training (see below).

**Procedure**

**Warmup.** The warmup was the same as in Study 1.

**Training phase.** In the training phase children learned to throw away a chest, and that after doing so they received the contents of all remaining chests.

***Training phase 1: Single chest demonstration.*** One single-chest demonstration introduced the child to the action of throwing away a chest. A single chest appeared on the screen. The experimenter explained that in this game, the child would pick one chest to throw away, and then win any coins left behind. The experimenter demonstrated throwing a chest away by clicking on the chest, causing it to fade out.

***Training phase 2: Two chests*.** Next, the child practiced throwing away an empty chest in order to win all coins that remained. Two chests appeared on the screen. The chests opened, a coin entered one of the chests, and the chests closed. The child was asked which chest they wanted to throw away. The chosen chest faded out; the other chest opened to reveal its contents. All children saw at least two trials.

***Training phase 3: Three chests*.** The Three-chest training trial encouraged the child to maximize the number of coins they received. Three chests appeared on screen, equally-spaced. The chests opened, a coin went into one of the chests, and the chests closed. Then the two empty chests opened, a coin went into one of them, and the chests closed. The child was asked which chest they wanted to throw away. The chosen chest faded out, and the remaining two chests revealed their contents in turn.

***Training phase 4: Flag training*.** The flag training trial familiarized children with flag occluders. Two chests appeared on the screen. Two flags appeared, one occluding each chest, with a gap between the flags. A coin descended behind one of the occluders, and the occluders disappeared, revealing only the two chests. The coin had to be inside one of the chests, and since there was a gap between the flags, children could infer which chest it was in. The child was asked which chest they wanted to throw away. The chosen chest faded out, and the remaining chest opened to reveal its contents.

***Training phase 5: Certainty-scaffolding demonstration*.** Penultimately, one demonstration trial encouraged children to attend to their own certainty. Three equally-spaced chests were occluded simultaneously with a single large pirate flag, and a coin went behind the flag. The experimenter told the child that the computer is going to hide a coin in one of the chests.

The flag was removed to show the three chests, each closed and hiding its contents, and the experimenter explained that he could not be sure where the coin was. This procedure was repeated two more times. After the third coin, the experimenter explained that he now *could* be sure where the coins were, as there had to be one coin in each chest.

**Test phase**. Two sets of chests appeared: a singleton and a pair (main text, Fig. 1). One set was occluded and a coin was hidden there, and the occluder disappeared. Then the other set was occluded and a second coin was hidden there, and the occluder disappeared. The experimenter reminded the child to try to win two coins. The child was then asked to pick a chest to throw away. The chosen chest faded out, and the remaining two chests opened to reveal their contents. Children saw eight trials. Three variables were counterbalanced between participants: the location of the singleton chest on the first trial (L/R), the positioning of the coin in the pair (LRRLRLLR / RLLRLRRL), and the side where coins were hidden first (L/R). The side (L/R) where coins were hidden first was constant within participants across trials.. The side of the singleton chest (L/R) swapped every trial.

**Study 3 (Preregisteration at https://aspredicted.org/aq2ct.pdf): Throw Away and Pick 1 of 2**

**Participants**

Participants were 24 3-year-old children (mean = 3.63, range = 3.04 to 3.96, 17 female). Nine additional children were excluded: three failed to complete the study, five made consecutive errors in the three chests training, and one made consecutive errors in the two chests training (see below). Exclusion criteria were identical to Study 2.

**Procedure**

**Warmup.** The warmup was the same as in Studies 1 and 2.

**Training phase.** In the training phase children learned to throw away a chest, and then to pick a chest to open.

***Training phases 1-3: Single chest demonstration, Two chests, and Flag training.*** All of these training trials were the same as in Study 2, except that when the chest was thrown away, a red X appeared to mark its former location.

***Training phase 4: Three chests.*** The purpose of the final training trial was to teach the child to throw away an empty chest and then open a chest that has a coin in it. Three equally-spaced chests appeared on screen. The chests opened, a coin went into one of the chests, and the chests closed again. The experimenter invited the child to throw away a chest. The selected chest faded out and a red X appeared in its place. Then the experimenter asked the child to pick a chest to open, so they could get what was inside, and reminded the child to open the treasure chest with the gold coin in it. The selected chest opened to reveal its contents.

**Test phase.** Two sets of chests appeared: a singleton and a pair (main text, Fig. 1). One set was occluded and a coin was hidden there, and the occluder disappeared. Then the other set was occluded and a second coin was hidden there, and the occluder disappeared. The experimenter asked the child which treasure chest they wanted to throw away. The selected

chest faded out. A red X appeared in its place to remind the child of the structure of the hiding phase: two chests in one set and only one chest on the other. Then the experimenter asked the child which chest they wanted to open. The selected chest opened and revealed its contents. Children saw eight trials. Three variables were counterbalanced between participants: the location of the singleton chest in the first trial (L/R), the side where coins were hidden tbdfirst in the first trial (L/R) and the positioning of the coin in the pair (LRRLRLLR / RLLRLRRL). The side of the singleton chest (L/R) and the side occluded first (L/R) swapped every trial.

**Statistical methods**

Data were analyzed with Bayesian random intercept GLMMs using the default weakly informative priors.The grouping variable in every model is participant id. All models were dummy coded. Below, response and predictor variables are specified when each model is introduced. Models were fitted with rstanarm (3). Emmeans (4), bayestestR (5) and tidybayes (6) generated posteriors. All credible intervals (CI) are 95% highest density posterior intervals, generated with emmeans. Model diagnostics appear in the online repository at dataverse.harvard.edu/tbd.

***Statistical Background***

Binary logistic models estimate the population mean log odds of a 1 response for each combination of predictors. In a Bayesian binary logistic model, the model estimate is not a point, but rather a distribution that captures the appropriate uncertainty about what the population mean is, given the data and the prior. Point estimates are typically provided by a measure of central tendency for that distribution. We have chosen to report the median as a measure of central tendency for all distributions, as the median tends to be more robust than the mean and maximum a posteriori (5). The maximum a posteriori is the continuous analog of the mode.

In Figure 2B (main text) posteriors are visualized as densities, the continuous analog of a histogram. The axes have been flipped so that the x-axis displays density (the continuous analog of count). The y-axis is a range of hypotheses regarding the probability of a wise decision. The area enclosed by the curve is 1. To evaluate a set of hypotheses about the probability of some event, e.g. the probability that children pick wisely more than 67% of the time on the throw away tasks, one calculates the proportion of the area enclosed by the curve associated with that set of hypotheses. In the example at hand, we would calculate the proportion of the area enclosed by the curve that is above .67 on the y-axis. To evaluate the probability of a point hypothesis, a Region of Practical Equivalence (ROPE) is defined around that hypothesis. This interval should be small enough that all points within that range are, for any practical purposes, equivalent to the hypothesis of interest. For example, to evaluate the probability that children pick the target cup 50% of the time on Pick 1 of 2, we will calculate the proportion of the area enclosed by the curve associated with the interval [.49, .51]. To evaluate the relative probability of this hypothesis, we divided the entire hypothesis space into 1000 discrete hypotheses, and calculated the probability of a ROPE of the same size around each of those 1000 hypotheses. We can then rank these ROPEs by their probability, and measure the relative probability of a given hypothesis by its rank: high ranking ROPEs have high relative probability, and low ranking ROPEs have low relative probability.

When the probability of an outcome is the same in two trial types (for example, if the probability of picking the singleton chest is .60 in both Pick 1 of 3 and Pick 1 of 2), then the odds of that outcome is also the same in both trial types (in this case, the odds of picking the singleton chest is 1.5 in both trial types). The ratio of those two odds is 1, and the log of that odds ratio is 0: no difference. A positive log odds ratio indicates greater odds in the numerator of the ratio than in the denominator. For example, if the probability of throwing away from the pair in Throw Away (Study 2) is .9 (odds = 9), and the probability of picking the singleton in Pick 1 of 3 is .6 (odds = 1.5), then the log of the odds ratio 9 / 1.5 is positive (in this example, it is about 1.79). Similarly, a negative log odds ratio indicates greater odds in the denominator. We will report contrasts as log odds ratios. We will always specify the numerator and denominator, e.g., "median log OR, Throw Away (Study 2) / Pick 1 of 3: 1.79". For all contrasts reported in this paper, a log odds ratio of ±0.52 can be considered a small effect, ±1.24 is medium, and ±1.90 is large (Chen et al. 2010).

***Comparisons to chance***

The main model predicted the probability of a wise decision from trial type, a factor with four levels: Pick 1 of 3 (Study 1), Throw Away (Study 2), Throw Away (Study 3), and Pick 1 of 2 (Study 3). For our first analysis we compared the estimated probability of making a wise decision in each trial type to chance. Chance was established by dividing the number of target cups by the total number of cups. We calculated the proportion of the posterior that was greater than these chance values in each trial type. In Pick 1 of 3 (Study 1), the entire posterior was greater than .33. In Throw Away (Study 2), the entire posterior was greater than .67. In Throw Away (Study 3), 99.98% of the posterior was above .67. There are two important conclusions from these results. First, children are not merely picking chests at random in any of these three trial types. Second, these results speak against the hypothesis that children deploy the low level strategies under discussion, as this hypothesis predicts that performance will be worse than chance on the Throw Away trial types.

In contrast with the first three trial types, in Pick 1 of 2 (Study 3), only 57.49% of the posterior was above .50, which is chance on this trial type (since there are only two cups to choose between). The median of this distribution was .51, 95% CI [.41, .62]. The probability that the population mean is .50 was evaluated by taking the interval [.49, .51] as a Region of Practical Equivalence (ROPE). The probability of this region was .148. By comparison, the probability of the ROPE [.50, .52] around the median, i.e., one of the most likely intervals of that width, is also .148. We divided the entire hypothesis space into 1000 point hypotheses, and calculated the probability of a ROPE of the same size around each of those 1000 hypotheses. We found that the interval [.49, .51] had higher probability than 99.5% of these intervals. Indeed, of the hypotheses within the 95% CI–that is, among the hypotheses that we would not reject on a frequentist analysis–the hypothesis that the population mean was .50 had higher probability than 99% of hypotheses.

In the first 3 trial types, we can have high confidence that the population mean is greater than chance. By contrast, in the last trial type, the hypothesis that the population mean is .50 (chance) is one of the highest probability hypotheses. Of course, a population mean of .50 could

come about in many different ways; for example, children could be picking chests at random (i.e., chance behavior), deploying minimal representations of possibility, or deploying the low level strategies under discussion. But children are not picking chests at random in any of the other trial types, even the same children in Study 3. Also, they are not deploying low level strategies in either of the Throw Away trial types. The full pattern of data prefers the hypothesis that children deploy minimal representations of possibility.

We now present three additional analyses. The first two differentiate among the three hypotheses depicted in Figure 2A, hypotheses that might explain the non-random behavior (60% wise choices) on Pick 1 of 3 tasks, including Study 1. First, we evaluate the differential predictions the three hypotheses make about the relative probabilities of wise decisions across trial types. Second, we turn to the quantitative predictions concerning probability of wise decisions. Third, we analyze the distribution of individual participants' proportion wise decisions in Study 1. The observed mean performance rate among 3-year-olds over many Pick 1 of 3 studies is around 60%, and there are many ways to arrive at this mean. We analyze the proportion of correct responses across participants. We test whether the observed distribution is much more likely generated from a 80%/20% mixture of children who deploy minimal representations of possibility/children who deploy possibility concepts, respectively, or from a 60%/40% mixture of children who guess at random/children who deploy possibility concepts, respectively.

**I. *Different Hypotheses Make Different Predictions about Relative Probabilities***

With respect to the relative probabilities of wise decisions, the hypothesis that most 3-year-olds deploy minimal representations of possibility predicts that performance on the Pick 1 trial types will be worse than performance on the Throw Away trial types (Figure 2A). The other two hypotheses predict no differences across the four levels of the predictor (Figure 2A; all 100% if children deploy possibility concepts; all 50% if children deploy low-level strategies). Studies 1 and 2 provide a between-participants measure of the difference between Pick 1 and Throw Away trial types; Study 3 provides a within-participants measure of that difference. In both cases children were more likely to maximize their expected reward on the Throw Away trial types than on the Pick 1 trial types (median log OR, Throw Away (Study 2) / Pick 1 of 3: 1.60, 95% CI [0.87, 2.36]; Throw Away (Study 3) / Pick 1 of 2: 1.41, 95% CI [0.91, 1.94]). These effects are most likely medium sized (7); neither of the 95% CIs include 0. Only the hypothesis that children deploy minimal representations of possibility predicts the full pattern of results. The other two hypotheses predict no difference, at least in their purest form, unsupplemented by additional assumptions.

However, the hypothesis that all 3-year-olds are attempting to deploy possibility concepts can be supplemented with assumptions to explain some of the observed differences. For example, perhaps the presence of 3 chests introduces noise at the time of planning a choice. This noise would yield worse performance on Pick 1 of 3 than on Throw Away (because there are two distractors vs one, respectively). Another auxiliary assumption that might generate the observed data is that despite having possibility concepts and appreciating all the possibilities, many 3-year-olds guess entirely at random (see also section III below). Both of these hypotheses

predict worse performance on Pick 1 of 3, where chance is 33%, than on the Throw Away trial types, where chance is 67%.

The results from Pick 1 of 2 speak against both of these auxiliary assumptions. Each predict that performance should be better on Pick 1 of 2 than from Pick 1 of 3, as there are two distractors in Pick 1 of 3 and only one distractor in Pick 1 of 2, and chance is lower in Pick 1 of 3 (33%) than in Pick 1 of 2 (50%). Contrary to the prediction that performance on Pick 1 of 2 would be better than that on Pick 1 of 3, it is most likely that performance is slightly worse (median log OR, Pick 1 of 2 / Pick 1 of 3: -0.43, 95% CI [-1.16, 0.20]). This log odds ratio does not reach the rule of thumb cut-off for a small effect, but more importantly, is in the wrong direction. The probability that children are even *slightly better* on Pick 1 of 2 than Pick 1 of 3 (i.e., a log odds ratio of 0.52 or greater) is only .003.

**II. *Quantitative Predictions of the Minimal Representation of Possibility Hypothesis***

The above analyses provide strong warrant to rule out the hypotheses that children deploy the low-level strategies under discussion and that they primarily deploy possibility concepts in this task. We turn now to the quantitative predictions of the hypothesis that children deploy minimal representations of possibility (Figure 2A). Children who deploy minimal representations of possibility should always pick wisely in the Throw Away trial types, but should only pick wisely half of the time in the Pick 1 of 3 and Pick 1 of 2 trial types. Three of these predictions are not precisely born out. In Pick 1 of 3 the modeled probability of choosing wisely was 61%, not 50%. In Throw Away (Study 2) the modeled probability was 89%, not 100%. In Throw Away (Study 3) the modeled probability was 81%, not 100%. We first discuss these three departures, and then discuss why no such departure is observed in Pick 1 of 2.

*Performance on Throw Away tasks is not 100%*

Performance on the Throw Away trials was not 100%. In Study 2 it was 89%; in Study 3 it was 81%. Of course, some noise is inevitable in studies with 3-year-olds. Sources of noise include momentary inattention, refusal to play the game the experimenter has established, and many others. Moreover, there is some pragmatic oddness to the Throw Away task in Study 3. In contrast to Study 2, where one gets the contents of *all* of all the chests that remain after one is thrown away, throwing a chest away has no purpose in Study 3. The participant would get the same result if they simply opened the desired chest. Though the difference between Throw Away (Study 2) and Throw Away (Study 3) is small, and the 95% CI includes 0 (median log OR, Throw Away (Study 3) / Throw Away (Study 2): -0.62, 95% CI [-1.36, 0.07]), there may be some small decrease in performance that is not due entirely to noise.

*Performance on Pick 1 of 3 is not 50%*

Three-year-olds pick wisely 61% of the time on Pick 1 of 3 (Study 1), not 50% as depicted in Figure 2A. This departure was expected, given existing data. One explanation for this finding is that minimal representations of possibility underlie the performance of most 3-year-olds, and that the construction of possibility concepts begins between ages 3 and 4. If this is correct, then we might expect older 2-year-olds to exhibit the predicted 50% level of responding on Pick from 3. We might also expect the difference between older 2-year-olds and 3-year-olds to be

small. To assess this, we assembled data from existing Pick 1 of 3 studies with older 2-year-olds and 3-year-olds. To prefigure the results: We found that the data are highly replicable within both age groups across all existing studies, that older 2-year-olds pick wisely about half of the time, and 3-year-olds are only slightly better.

First we assembled all existing data on Pick 1 of 3 tasks with older 2-year-olds and 3-year-olds (1, 2, current data) and fit a model predicting the probability of choosing wisely from Study, age group, and their interaction. The rate of rational choice of the singleton chest was highly replicated in both age groups across the three studies. For older 2-year-olds (30 months to 36 months) the estimated probability of choosing the singleton was: Mody & Carey .46, Grigoroglou et al. .48. The difference between these two studies was effectively 0 (median log OR, Mody & Carey / Grigoroglou et al.: -0.07, 95% CI [-0.83, 0.68]). For 3-year-olds the estimated probability of choosing the singleton was: Mody & Carey 2016: .61; Grigoroglou et al. 2019: .62; Study 1: .61. In all three pairwise comparisons, the median difference between studies was effectively 0 (median log OR, Study 1 / Mody & Carey 2016: -0.01, 95% CI [-0.66, 0.65]; Study 1 / Grigoroglou et al. 2019: -0.03, 95% CI [-0.72, 0.62]; Grigoroglou et al. 2019 / Mody & Carey 2016: 0.03, 95% CI [-0.67, 0.76]). A difference between -0.1 and 0.1 can be considered, for all practical purposes, no difference at all (8). The data are highly replicable in both age groups. This justifies collapsing the data across these studies in further analyses.

Under the hypothesis that a small handful of 3-year-olds have developed possibility concepts, while all older 2-year-olds deploy minimal representations of possibility, older 2-year-olds should pick wisely 50% of the time; 3-year-olds' performance should be only slightly better. To test this, we fit a model predicting the probability of a wise decision from age group alone. For older 2-year-olds, the median probability of choosing the target is .47, 95% CI [.38, .57]. Performance was better than chance (probability > .33 = .998). Since the 95% CI includes .5, the hypothesis that the population mean for older 2-year-olds is .5 cannot be ruled out. For a more nuanced analysis of the probability that older 2-year-olds pick the target 50% of the time, we defined a ROPE around .5 as the interval [.49, .51]. The probability of this interval is .14. For comparison, the probability of the ROPE [.46, .48] around the median–one of the highest probability hypotheses–is .16. We calculated similar ROPEs for 1000 hypotheses divided uniformly over the entire hypothesis space. The probability of the interval [.49, .51] was higher than 94% of these intervals. More conservatively, we compared the hypotheses that were inside the 95% CI (i.e., the hypotheses that a frequentist analysis cannot distinguish between). We found that the probability of the interval [.49, .51] was higher than the probability of 69% of these intervals. Not only can the hypothesis that older 2-year-olds pick the target 50% of the time not be ruled out; given the current data and our priors, this hypothesis is one of the highest probability hypotheses, as predicted if almost all older 2-year-olds deploy minimal representations of possibility.

Moreover, the difference between older 2-year-olds and 3-year-olds was not large. The most likely effect of age group was small (median log OR, 3-year-olds / older 2-year-olds: 0.56,

95% CI [0.10, 1.03]. It is likely that there is some improvement with age, as the 95% CI does not include 0. It is unlikely that the effect is even medium sized, as the 95% CI does not include 1.24.

Thus, as predicted by the hypothesis that all older 2-year-olds and most 3-year-olds deploy minimal representations of possibility, and that a small handful of 3-year-olds deploy possibility concepts, the estimated population mean for older 2-year-olds is about .5, and 3-year-olds are only slightly better. Both of these predictions are supported by the data.

*Pick 1 of 2 is almost exactly 50%*

Finally, we discuss why 3-year-olds' performance in Pick 1 of 2 is almost exactly 50%, especially if a small proportion of 3-year-olds deploy possibility concepts, as demonstrated by the highly systematic 60% wise decisions in this age group on Pick 1 of 3. In fact, we preregistered the prediction that performance on Pick 1 of 2 would be about 60%, and the same as performance on Pick 1 of 3. First we note that the difference between Pick 1 of 3 and Pick 1 of 2 is small and the 95% CI includes 0 (median log OR, Pick 1 of 2 / Pick 1 of 3: -0.43, 95% CI [-1.16, 0.20]); there may be nothing to explain here. Also, recall that there was a similar decrease in performance from Throw Away (Study 2) to Throw Away (Study 3): median log OR, Throw Away (Study 3) / Throw Away (Study 2): -0.62, 95% CI [-1.36, 0.07]. We proposed above that this decrease (if it exists) may be due to the pragmatic strangeness of throwing away a chest before picking one to open. The same pragmatic strangeness may have caused the small drop in performance from Pick 1 of 3 to Pick 1 of 2 (if it exists). Children may simply have been confused about why they were throwing away a chest before choosing one to open. One idea for how to test this proposal would be to treat the Throw Away (Study 2) procedure as a training process for the Study 3 procedure. If children have substantial practice throwing away a chest in a context where throwing away a chest is pragmatically sensible, then adding the choice of which chest to open might be less demanding. This could result in improved performance on both the Throw Away trials prior to a choice and on the Pick 1 of 2 trial type.

**III. Distribution of individual participants' proportion wise decisions on Study 1**

In our explanation for why 3-year-olds' performance on Pick 1 of 3 is not 50%, we suggested that the population we sampled from is composed of two groups of children: one group who deploy minimal representations of possibility (thereby choosing wisely half of the time), and another group who deploy possibility concepts (thereby choosing wisely on every trial). An alternative hypothesis is that observed performance arises from a mixture of children who guess randomly (thereby choosing wisely a third of the time) and children who deploy possibility concepts. Notice that this alternative hypothesis is not one we have considered as of yet in this manuscript. On this hypothesis, children increasingly deploy modal concepts over the ages of 2 ½ to 4 or 5. Children who do not deploy modal concepts deploy neither minimal representations nor low level strategies, but rather choose randomly among the three containers. Throughout the paper we emphasized that chance-level performance is not observed on Pick 1 of 3. But we should also test whether the observed 60% performance rate on the Pick 1 of 3 measure is due to a mixture of children deploying modal concepts and children merely guessing among the visible cups.

The observed mean in the current study (approximately 60% choice of the singleton) yields an estimate for how these populations are most likely mixed under each hypothesis. To simplify, we assume that each child deploys a single strategy across trials: Children deploying possibility concepts choose wisely on every trial, children deploying minimal representations choose between the two chests they represent as containing coins at random on every trial, and children who are guessing choose among the three chests randomly on every trial. If the population is a mixture of children who deploy minimal representations of possibility and children who deploy possibility concepts, the most likely mixture is 80%/20%: (.8 * .5) + (.2 * 1) = .60. If the population is a mixture of children who guess at random and children who deploy possibility concepts, the most likely mixture is 60%/40%: (.6 * .33) + (.4 * 1) = .60. We can then use exact multinomial goodness-of-fit tests to evaluate these two hypotheses against the observed data by comparing the observed distribution of trials correct within individuals to the expected distribution of trials correct within individuals under each hypothesis.

Expected distributions were calculated by first calculating the expected number of children deploying each strategy. For example, under the hypothesis of a 60%/40% mixture of children who choose at random and children who deploy possibility concepts, we expect .6 * 24 = 14.4 children who guess randomly. Next we calculated the probability of having 0 through 4 successes using the binomial density function: $P_x = \binom{n}{x} p^x (1-p)^{n-x}$ where x is a number of successes, n is the number of trials, p is the probability of success on a single trial, and $P_x$ is the probability of having x-many successes. We multiplied this vector of probabilities by the expected number of children: <.2, .4, .3, .09, .01> * 14.4 = <2.8, 5.7, 4.2, 1.4, .18>. We then added the related vector for the other probability in the mixture: For example, we expect .4 * 24 = 9.6 children who deploy possibility concepts and always pick the singleton chest; <0, 0, 0, 0, 1> * 9.6 = <0, 0, 0, 0, 9.6>; added to <2.8, 5.7, 4.2, 1.4, .18> yields <2.8, 5.7, 4.2, 1.4, 9.78>. This final vector is the expected distribution if the population is a 60%/40% mixture of children who choose at random and children who deploy possibility concepts, respectively. Applying the same process to calculate the expected distribution if the population is an 80%/20% mixture of children who deploy minimal representations of possibility and children who deploy possibility concepts yields <1.2, 4.8, 7.2, 4.8, 6> as expected distribution.

In the observed Pick 1 of 3 data, 0% of children choose the singleton 0 of 4 times, 21% 1 of 4 times, 37% 2 of 4 times, 21% 3 of 4 times, and 21% 4 of 4 times; see small dots in Figure 2b, Study 1, main text. The observed distribution is not significantly different from the distribution that is expected if the population is an 80%/20% mixture of minimal representers and children who deploy possibility concepts (observed distribution: <0, 5, 9, 5, 5>, expected distribution: <1.2, 4.8, 7.2, 4.8, 6.0>, exact multinomial test: p = .90). The observed distribution is significantly different from the distribution that is expected if the population is a 60%/40% mixture of random guessers and children who deploy possibility concepts (observed distribution: <0, 5, 9, 5, 5>, expected distribution: <2.84, 5.69, 4.27, 1.42, 9.78>, exact multinomial test: p =.001).

We repeated these analyses on all existing data sets of 3-year-olds in the 3-containers task to evaluate the most likely mixtures. We combined the existing datasets (1, 2), eliminating participants who did not see a full complement of 3 trials, yielding a sample of 46 3-year-olds. In the observed data, 9% chose the singleton 0 of 3 times, 30% 1 of 3 times, 35% 2 of 3 times, and 26% 3 of 3 times. The observed distribution is not significantly different from the distribution that is expected if the population is an 80%/20% mixture of children who deploy minimal representations of possibility and children who deploy possibility concepts (observed distribution: <4, 14, 16, 12>, expected distribution: <4.6, 13.8, 13.8, 13.8>, exact multinomial test: $p = .91$). The observed distribution is significantly different from the distribution that is expected if the population is a 60%/40% mixture of children who guess at random and deploy possibility concepts, respectively (observed distribution: <4, 14, 16, 12>, expected distribution: <8.2, 12.3, 6.1, 19.4>, exact multinomial test: $p < .001$).

Next, we present the analysis of existing data from older 2-year-olds (1, 2). After removing participants who did not see a full complement of 3 trials, we have $n = 41$. Because the median probability of choosing the singleton in this age group is .47 (see above), we need to recalculate the most likely mixtures for each hypothesis. If the population is a mixture of children deploying minimal representations of possibility and children deploying possibility concepts, it is most likely that the population is 100% children who deploy minimal representations of possibility ($1 * .5 + 0 * 1 = .5$). If the population is a mixture of children who guess at random and children who deploy possibility concepts, the most likely mixture is 79.5% children who guess at random and 20.5% children who deploy possibility concepts and always choose the singleton ($.795 * .33 + .205 * 1 = .47$). Assuming these mixtures, we can evaluate the probability of the observed distribution of proportion correct responses against the expected distribution under each hypothesis.

In the observed data, 7% chose the singleton 0 of 3 times, 51% 1 of 3 times, 37% 2 of 3 times, and 5% 3 of 3 times. The observed distribution is not significantly different from the distribution that is expected if all older 2-year-olds deploy minimal representations of possibility (observed distribution: <3, 21, 15, 2>, expected distribution: <5.1, 15.4, 15.4, 5.1>, exact multinomial test: $p = .23$). The observed distribution is significantly different from the distribution that is expected if 79.5% of older 2-year-olds guess randomly while 20.5% deploy possibility concepts (observed distribution: <3, 21, 15, 2>, expected distribution: <9.7, 14.5, 7.2, 9.6>, exact multinomial test: $p < .001$).

In every group, the data are unlikely under the hypothesis that the population is a mixture of children who guess randomly and others who deploy possibility concepts. The data are not unlikely under the hypothesis that the population is a mixture of children who deploy minimal representations of possibility and children who deploy possibility concepts.

**Excluded Data**

In the analysis of the Pick 1 of 2 data, we excluded trials where children had thrown away the singleton chest. This is because the question, “Did they choose the singleton?” is not defined when the singleton was thrown away. This was 21% of trials in Study 3, which raises an important

concern. Throwing away the singleton might indicate a failure to understand the task, and perhaps a large part of the data that was actually analyzed is also contributed by children who did not understand the task. There are two reasons to doubt that this is so. First, performance is well above chance on both Throw Away measures. Second, children deploying minimal representations of possibility in Study 3 represent that the singleton contains a coin and which chest from the pair contains a coin. If these beliefs were true, there would be nothing irrational about throwing away the singleton, as one could simply pick the remaining chest that holds a coin in the second phase. In Study 2, in contrast, after they throw away one of the chests, they get all the remaining chests. This provides motivation to throw away an empty chest, and they throw away the singleton chest less than in Study 3 (median log OR, Throw Away (Study 3) / Throw Away (Study 2): -0.62, 95% CI [-1.36, 0.07]; see Section II above).

**References**

[1] Mody, S., & Carey, S. (2016). The emergence of reasoning by the disjunctive syllogism in early childhood. *Cognition*, *154*, 40-48.

[2] Grigoroglou, M., Chan, S., & Ganea, P. A. (2019). Toddlers' understanding and use of verbal negation in inferential reasoning search tasks. *Journal of experimental child psychology*, *183*, 222-241.

[3] Goodrich B, Gabry J, Ali I & Brilleman S. (2020). rstanarm: Bayesian applied regression modeling via Stan. R package version 2.21.1. https://mc-stan.org/rstanarm.

[4] Lenth, R. (2021). emmeans: Estimated Marginal Means, aka Least-Squares Means. R package version 1.7.1-1. https://CRAN.R-project.org/package=emmeans.

[5] Makowski, D., Ben-Shachar, M., & Lüdecke, D. (2019). bayestestR: Describing Effects and their Uncertainty, Existence and Significance within the Bayesian Framework. Journal of Open Source Software, 4(40), 1541. doi:10.21105/joss.01541.

[6] Kay, M. (2022). tidybayes: Tidy Data and Geoms for Bayesian Models. R package version 3.0.2, http://mjskay.github.io/tidybayes/, doi:10.5281/zenodo.1308151.

[7] Chen, H., Cohen, P., & Chen, S. (2010). How big is a big odds ratio? Interpreting the magnitudes of odds ratios in epidemiological studies. *Communications in Statistics—simulation and Computation®*, *39*(4), 860-864.

[8] Cohen, J. (2013). *Statistical power analysis for the behavioral sciences*. Academic press.
